# Complete Genome of Salmonella enterica Serovar Typhimurium T5-Like Siphophage Stitch

**DOI:** 10.1128/genomeA.01435-14

**Published:** 2015-02-05

**Authors:** James M. Grover, Adrian J. Luna, Thammajun L. Wood, Karthik R. Chamakura, Gabriel F. Kuty Everett

**Affiliations:** a Center for Phage Technology, Texas A&M University, College Station, Texas, USA; b Department of Biochemistry and Molecular Biology, Pennsylvania State University, University Park, Pennsylvania, USA

## Abstract

Salmonellosis, caused by Salmonella, is a leading cause of food poisoning worldwide. With the continuing rise of bacterial antibiotic resistance, efforts are focused on seeking new approaches for treatment of bacterial infections, namely, bacteriophage therapy. Here, we report the complete genome of S. Typhimurium siphophage Stitch.

## GENOME ANNOUNCEMENT

Salmonellosis is caused by serovars of the Gram-negative bacterium Salmonella enterica and is a leading cause of food poisoning (1, 2). With reports of antibiotic resistance on the rise, bacteriophage therapy presents an appealing alternative for treatment of bacterial infections (3, 4). Before a phage can be used in a clinical setting, much research is required to define various aspects of the phage’s life cycle (5). To that end, we report here the complete genome of S. enterica serovar Typhimurium T5-like siphophage Stitch.

Stitch was isolated from a sewage sample collected in College Station, TX, USA. Phage DNA was sequenced using 454 pyrosequencing at the Emory GRA Genome Center (Emory University, GA, USA). Trimmed FLX Titanium reads were assembled to a single contig using the Newbler assembler, version 2.0.01.14 (454 Life Sciences), at default settings. Additional sequencing was performed at the Genomic Sequencing and Analysis Facility at the University of Texas (Austin, TX, USA) to give a single contig at 80.9-fold coverage. Genes were predicted using GeneMarkS (6) and corrected using software tools available on the Center for Phage Technology (CPT) Galaxy instance (https://cpt.tamu.edu/galaxy-public/). Transmission electron microscopy was performed at the Microscopy and Imaging Center at Texas A&M University.

Stitch shares 62% sequence identity with Enterobacteria phage T5 (NC_005859), as determined by Emboss Stretcher analysis (7). The differences between the two phages occur largely in hypothetical conserved and novel genes. Stitch has a unit genome of 113,943 bp with 165 predicted coding sequences. A 9,982-bp-long terminal repeat was annotated using the PAUSE method (https://cpt.tamu.edu/pause) on raw sequencing data. Twenty-eight tRNA genes were identified in Stitch compared to the 16 tRNA genes present in T5.

As a T5-like phage, the genome of Stitch can be divided into several gene clusters. Proteins for host shutdown (presumably pre-early genes) were found, including 5′-deoxyribonucleotidase, A1, and A2 (8, 9). The early gene region encodes proteins whose functions relate to DNA metabolism, replication, regulation, and lysis. The late gene cluster consists of morphogenesis genes. As with T5, a tail protein was found present in a noncanonical location among the major capsid protein, the prohead protease, portal, and large terminase (8). Stitch also encodes the lytic conversion lipoprotein (Llp). The Llp binds to host FhuA to prevent the lysed cell from inactivating released progeny (10, 11). Stitch contains no HNH homing endonucleases compared to T5’s 9 homing endonucleases.

Stitch encodes a nicotinamide mononucleotide (NMN) transporter and an NMN adenylyltransferase that is found in T5-like phage EPS7 (NC_010583) but not in T5 itself. NMN adenylyltransferase catalyzes the biosynthesis of NAD^+^ and PPi from the condensation of NMN and ATP (12). As a T5-like phage, Stitch presumably has a nicked genome. It encodes A and B subunits of the NAD-dependent DNA ligase that are needed to seal the nicks and support genome replication (8). The NMN transporter and adenylyltransferase probably aids in efficient ligation and replication of the nicked genome.

### Nucleotide sequence accession number.

The genome sequence of phage Stitch was deposited in GenBank under the accession number KM236244.
